# Profil du facteur Von Willebrand dans la grossesse: étude descriptive chez 390 femmes enceintes au Maroc

**DOI:** 10.11604/pamj.2018.31.232.13138

**Published:** 2018-12-18

**Authors:** Mamad Hassane, Souad Benkirane, Youssef Motiaa, Fatima Dahmani, Mohammed Elkhorassani, Azlarab Masrar

**Affiliations:** 1 Laboratoire d'Hématologie, Équipe de Recherche en Hématologie, Faculté de Médecine et de Pharmacie, Université Mohammed V, Rabat, Maroc; 2 Laboratoire Central d'Hématologie, Centre Hospitalier Ibn Sina, Rabat, Maroc; 3 Service de Réanimation Médicale, Institut National d'Oncologie, Centre Hospitalier Ibn Sina, Rabat, Maroc; 4 Service d'Hématologie et d'Oncologie Pédiatrique, Centre Hospitalier Ibn Sina, Rabat, Maroc

**Keywords:** Hémostase, facteur Von Willebrand, maladie de Willebrand, grossesse, Hemostasis, Von Willebrand factor, Von Willebrand disease, pregnancy

## Abstract

Le facteur Von Willebrand (vWF) est une glycoprotéine qui joue un rôle important dans l'hémostase, dont son déficit quantitatif ou qualitatif induit la maladie de Willebrand (MV). Le but est de mettre le point sur la répartition des taux de vWF dans la population marocaine chez les femmes enceintes et sa variabilité en fonction du groupe sanguin ABO. Il s'agit d'une étude transversale qui a intéressé 390 femmes enceintes provenant de la région Rabat-Salé-Kenitra dont la taille de l'échantillon a été calculée sur la base d'une prévalence de 1% correspondant à la prévalence mondiale de la MW avec une marge d'erreur de 5% et un niveau de confiance de 95%. Trois cent dix-sept cas sur 390 femmes enceintes se sont révélés avec des taux élevés aux normes (> 160%) en vWF soit 81,28%. Les taux de facteur VIII (FVIII) varient en parallèle de façon significative p < 0,001 avec les taux de vWF avec un cœfficient de correlation (r) de *Pearson* à 0,597. La distribution des groupes sanguins A,B et O a eu une influence sur le taux de vWF avec une différence significative p < 0,001 entre les quatre groupes: niveau moyen le plus bas chez le groupe O (188,54±57,02), suivi par le groupe A (203,19±54,46), puis le groupe AB (219±38,95), et enfin le groupe B (221,15±48,63). Nos résultats confirment d'une part l'élévation des taux de vWF pendant la grossesse et d'autre part l'influence du groupe sanguin ABO sur les taux du vWF.

## Abstract (en)

Von Willebrand factor (vWF) is a glycoprotein which plays an important role in hemostasis. Its quantitative or qualitative deficit results in von Willebrand's disease (vWD). The study aims to update the current understanding of the distribution of vWF rates in Moroccan pregnant women and their variability in function of the ABO blood group. We conducted a cross-sectional study of 390 pregnant women from the Rabat-Salé-Kenitra region. Sample size was calculated on the basis of a prevalence of 1%, corresponding to the global prevalence of vWD with a margin of error of 5% and a confidence level of 95%. There were 317 cases (81.28%) of elevated vWF levels (> 160%) out 390 pregnant women. The levels of factor VIII (FVIII) varied in parallel in a significant way (p < 0.001) with the levels of vWF (Pearson's r 0,597). The distribution of the ABO blood groups has had an influence on the level of vWF with a significant difference (p < 0.001) between the four groups: the lowest average level in the group O (188,54±57,02), followed by group A (203,19±54,46), then group AB (219±38,95) and finally group B (221,15±48,63). Our results confirm on the one hand an elevation of the levels of vWF during pregnancy and on the other hand the influence of ABO blood group on the levels of vWF.

## Introduction

La maladie de Willebrand (MW) est une des plus fréquentes anomalies constitutionnelles de l'hémostase. Sa transmission est autosomique, généralement dominante. Elle est liée à une anomalie, soit quantitative, soit qualitative du facteur Von Willebrand (vWF). Le risque hémorragique est variable selon la sévérité de l'atteinte, mais les femmes présentent une symptomatologie clinique plus marquée en raison des risques supplémentaires d'hémorragies lors des menstruations, surtout lors des accouchements et en post-partum. La fréquence du déficit en vWF a pu être estimée de 1 à 2% de la population générale, mais la prévalence des formes symptomatiques est évaluée à 0,01% [1]. L'objectif de notre étude transversale et descriptive est de souligner la répartition des taux du vWF dans une population de femmes enceintes marocaines tout en insistant sur l'influence du groupe sanguin ABO sur les taux du vWF et en rapportant les cas de la maladie de Willebrand diagnostiquée chez cette population.

## Méthodes

Il s'agit d'une étude transversale et descriptive réalisée au Centre Hospitalier Ibn Sina Rabat en collaboration entre le laboratoire central d'hématologie, le centre de traitement de l'hémophilie et les urgences de la maternité Souissi. Cette étude a intéressé 390 femmes enceintes provenant de la région Rabat-Salé-Kenitra dont la taille de l'échantillon a été calculée sur la base d'une prévalence de 1% correspondant à la prévalence mondiale de la MW avec une marge d'erreur de 5% et un niveau de confiance de 95%. La fiche d'exploitation dument renseignée par le clinicien et complétée par le laboratoire contient les informations suivantes: les caractéristiques épidémiologiques: nom, prénom, âge; l'histoire hémorragique: personnels (mode, fréquence et intensité de saignement) et familiaux (consanguinité).

**Phase pré-analytique:** Les prélèvements sanguins sont effectués au service des urgences de la maternité Souissi par ponction veineuse dans des tubes sous vide contenant comme anticoagulant du citrate trisodique (0,109M). Le mélange sang/anticoagulant (9V/1V) est assuré immédiatement par retournements successifs et lents. L'acheminent des prélèvements par le centre de traitement d'hémophilie se fait à température ambiante (20°C +/- 2°C) dans un délai de moins de 2 heures. Au laboratoire central d'hématologie, les prélèvements subissent immédiatement une double centrifugation à 3000g pendant 15min. Par ailleurs, la congélation des échantillons plasmatiques à -80°C se fait par décantation en tubes remplis au tiers, bouchons à vis préconisés. Pour la décongélation, les échantillons congelés doivent être incubés dans le bain marie pendant 10 minutes à 37°C et laisser reposer pendant 10 min à température ambiante 20°C +/- 2°C, puis agitation par retournements ou vortex.

**Phase analytique:** Au sein du laboratoire, les tests d'hémostase sont réalisés par la méthode optique sur l'automate Sysmex CS-5100 et les réactifs Siemens. Les tests hématologiques réalisés sont: temps de céphaline avec activateur (TCA), taux de prothrombine (TP), temps de Quick (TQ), taux de fibrinogène (Fg), activité en vWF, taux du FVIII et groupage sanguin ABO-Rhésus (RH).

**Analyse statistique:** Les données ont été saisies et analysées à l'aide du logiciel SPSS 20.0 (Inc, chicago,Il). Les variables quantitatives ont été exprimées en moyenne ± écart type vue que la distribution des différentes variables est gaussienne. Les variables qualitatives ont été exprimées en effectif et pourcentage et leur comparaison a été réalisée par le test chi-2. Les variables quantitatives ont été comparées en utilisant une analyse de variance ANOVA One -Way Analysis of Varainc, avec un test post hoc par le test de Benferoni quand il existe une différence statistiquement significative à l'ANOVA. Le test de Pearson a été utilisé pour étudier la corrélation entre les variables quantitatives avec calcul du facteur r de corrélation. Une différence est considérée comme statistiquement significative quand le p < 0,05.

## Résultats

**Répartition des patients selon les tranches d'âge:** L'étude a porté sur 390 femmes, l'âge moyen est de 28,44 ans ±6,6 avec des extrêmes de 16 et 45 ans.

**Répartition des patients selon les tests d'hémostase:** Sur les 390 femmes enceintes, le taux de prothrombine (TP) est normal (70 à 100%) chez la quasi-totalité soit 99,75% sauf chez une seule patiente qui a un TP bas à 55,9%. Quant aux rapports temps de cephaline avec activateur (TCA), 373 avaient un ratio TCA normal (TCA patient / TCA témoin ≤ 1,2), soit 95,64% contre 17 patientes qui se sont révélées avec un ratio TCA allongé soit 4,36%. Les taux de fibrinogène n'étaient diminués chez aucune de nos patientes. Par ailleurs, 38,97% des taux étaient dans les normes (entre 2 à 4g/l) et 61,03% se sont révélés supérieurs aux valeurs usuelles. Selon les taux en facteur VIII, nos patientes étaient réparties en 2 patientes qui avaient un taux < 50%, soit 0,51%; 29 patientes étaient dans les normes (50 à 145%) soit 7,44% et 359 cas au-delà du seuil normal (> 145%), soit 92,05%. Tandis que les taux en vWF étaient diminués (< 50%) chez 3 patientes soit 0,77%, ceux compris dans les normes (50-160%) chez 70 patientes soit 17,95% alors que les taux élevés (>160%) chez 317 patientes soit 81,28%.

**Répartition des patientes selon la fréquence phénotypique ABO-RH:** Les résultats du groupage sanguin ABO montrent que le groupe O se trouve chez 42,56% des femmes, le groupe A chez 33,33%, le groupe B 17,44% et le groupe AB chez 6,67%. Pour le groupe sanguin RH, les patientes Rh positif représentent 92,82% et celles de Rh négatif sont à 7,18%.

**Figure 1:**
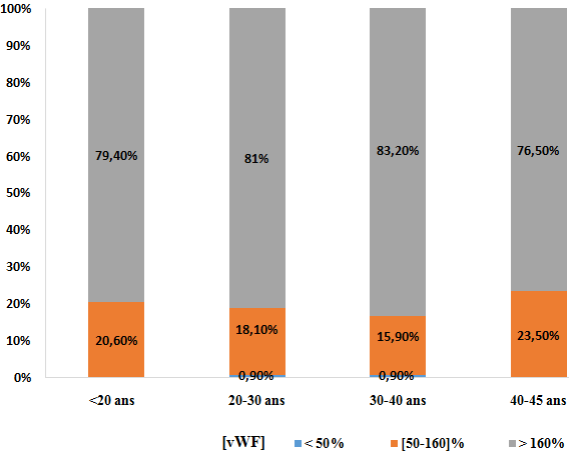
Répartition des taux de vWF en fonction des tranches d'âge

**Figure 2:**
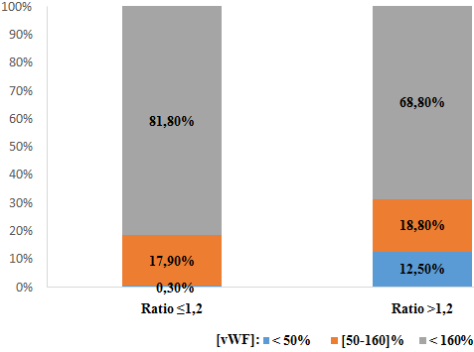
Répartition des taux de vWF en fonction du rapport TCA

**Figure 3:**
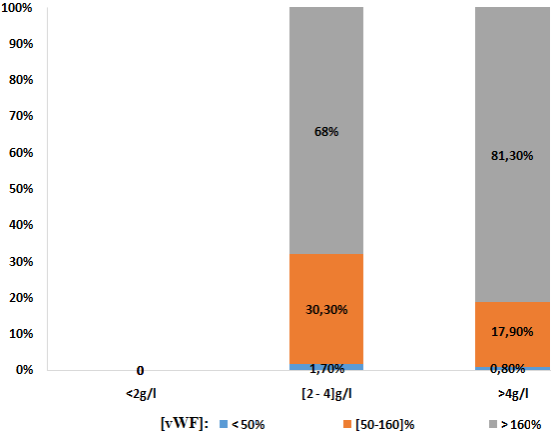
Répartition des taux de vWF en fonction des taux de fibrinogène

**Figure 4:**
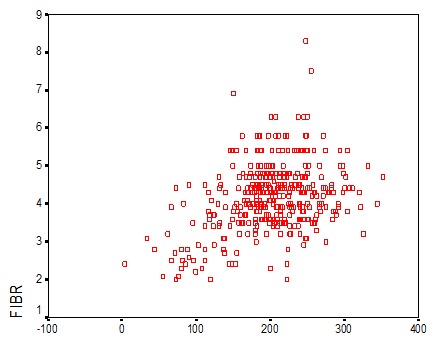
Corrélation entre les taux de vWF et fibrinogène

**Figure 5:**
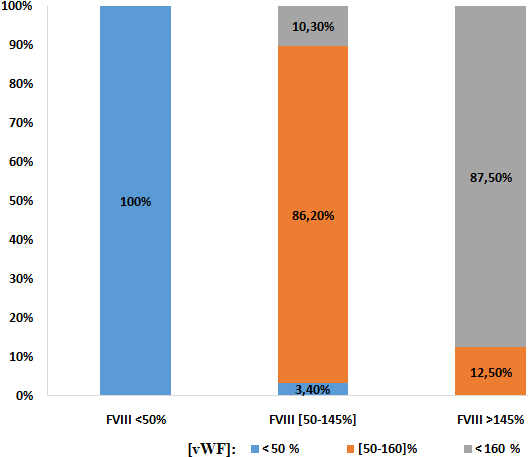
Répartition des taux de vWF en fonction des taux de FVIII

**Figure 6:**
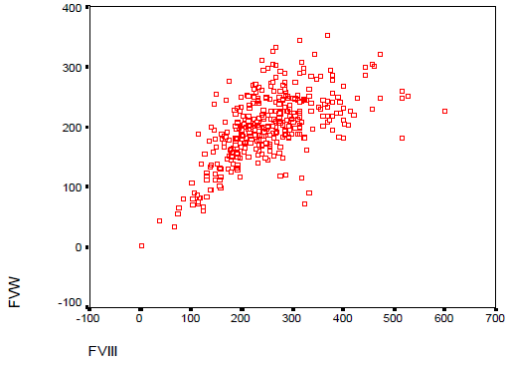
Corrélation entre les taux de vWF et FVIII

**Répartition des taux de vWF en fonction des autres paramètres:** On trouve qu'avec l'âge, les taux de vWF s'élèvent comme indiqué sur les histogrammes, dont les taux élevés sont occupés par 83,2% de la population en avançant dans l'âge (Figure 1). Sur les 390 femmes, une seule patiente avait un TP bas (< 70%) avec un taux de vWF normal. Pour les 389 patientes qui avaient un TP normal (70-100%): 1% avaient un taux de vWF diminué, 17,7% dans les normes et 81,5% se sont révélées avec des taux élevés. Pour un ratio TCA normal (≤ 1,2): une seule patiente soit 0,3% avec un taux bas en vWF, 17,9% dans les normes et 81,8% ont un taux supérieur à la normale. Pour un ratio TCA >1,2: 12,5% avec un taux vWF diminué, 18,8% dans les normes et 68,8% se sont révélés avec un taux élevé à la valeur normale (Figure 2). Le dosage du fibrinogène n'a révélé aucune valeur inférieure à la normale (2-4g/l).Pour des valeurs normales: 1,7% avaient un taux bas en vWF, 30,0% dans les normes, et 68% avec des taux supérieurs aux valeurs usuelles, tandis que pour les hausses valeurs en Fg (> 4g/l): 0,8% avaient un taux bas en vWF, 17,9% dans les normes et 81,3% avec des valeurs supérieures aux normes (Figure 3). Ainsi on trouve que les taux en fibrinogène varient en parallèle avec ceux en vWF (Figure 4). Toutes les patientes qui ont un taux de FVIII diminué (< 50%), ont un taux bas en vWF. Pour les valeurs normales en FVIII (50-145%): une patiente, soit 3,4%, avait une valeur diminuée en vWF, 86,2% dans les normes et 10,3% supérieures aux normes, et pour des taux élevés en FVIII (>145%): 12,5% dans les normes et 87,5% avec des taux élevés (Figure 5). Du même on trouve que les taux de FVIII varient en parallèle avec ceux du vWF (Figure 6). Selon les groupes du système ABO, les taux de vWF varient dans le sens croissant: groupe O < groupe A < groupe B < groupe AB. Par ailleurs, la même répartition est notée en fonction des taux en FVIII (Tableau 1).

**Tableau 1:** Répartition des taux de vWF et FVIII en fonction du groupe sanguin ABO

	B	AB	A	O	P value
**vWF(%)**	221,15±48,63	219±38,95	203,19±54,46	188,54±57,02	< 0,001
**FVIII(%)**	287,91±89,72	269,83±70,03	254,61±80,51	233,70±80,79	< 0,001

## Discussion

Notre étude a porté sur 390 femmes enceintes, l'âge moyen était de 28,44±6,6 ans avec des extrêmes de 16 et 45 ans, dont 58% représentait par la classe jeune âgée entre 20 et 30 ans. La plupart des facteurs d'hémostase augmentent pendant la grossesse, particulièrement le fibrinogène, le facteur VIII et le facteur de Willebrand (vWF) [2]. Sur les 390 femmes, le TP était normal (70 à 100%) chez la quasi-totalité soit 99,75% sauf chez une seule patiente avec un TP à 55,9%. Pour le rapport TCA patient / TCA témoin, 373 patientes avaient un ratio normal (≤ 1,2), soit 95,64% contre 17 patientes qui se sont révélées avec un ratio anormal soit 4,36%. Ces résultats concordent avec la littérature, l'état de la grossesse représente un terrain d'hypercoagulabilité connaissant l'augmentation de certains facteurs de coagulation. Cette augmentation est d'ailleurs responsable d'un net raccourcissement du TQ (TP) et du TCA chez toutes les femmes en fin de grossesse [3]. Les taux de fibrinogène chez nos patientes n'étaient pas diminués chez aucune de nos patientes, par contre les taux normaux (2 à 4g/l) étaient trouvés chez 38,97% des cas et 61,03% se sont révélés avec des taux supérieurs aux valeurs usuelles. Donc on note une nette élévation des taux de fibrinogène en cette situation de grossesse.

Nos patients étaient répartis selon les taux du facteur VIII comme suit: 2 patientes qui avaient un taux bas, soit 0,51% (< 50%), 29 patients inclus dans les normes (50 à 145%) soit 7,44%, et 359 cas au-delà du seuil normal (>145%), soit 92,05% de la totalité de l'échantillon étudié pour attendre un maximum de 599%. Ces taux concordent avec des études réalisées sur l'hémostase et la grossesse [2]. Les valeurs de vWF enregistrées pour nos patientes étaient diminuées pour 3 patientes soit 0,77% (< 50%), les taux en vWF étaient compris dans les normes (50-160%) pour 70 patientes soit 17,95%, tandis que ces valeurs se sont révélés élevés chez 317 patientes soit 81,28% (> 160%), exprimant l'état du terrain étudié. Tous ces résultats concordent avec la littérature [2]. Les taux de fibrinogène, FVIII et vWF augmentent progressivement au cours de la grossesse avec un taux de multiplication par deux pour le fibrinogène et le FVIII et par trois pour le vWF [4, 5]. Pour la fréquence phynotypique, d'une part on constate que les groupes du système ABO prédominent dans l'ordre décroissant suivant: groupe O, groupe A, groupe B et groupe AB. Le groupe O se trouve chez environ la moitié des personnes phénotypées (42,56%), le groupe A est presque deux fois supérieur (33,33%) au groupe B (17,44%), le groupe AB a la fréquence la plus faible (6,67%). D'autre part nous constatons une nette prédominance des sujets Rh positif (92,82%) par rapport aux sujets Rh négatif (7,18%) dans notre population étudiée. Nos résultats indiquent une fréquence des groupes A, B, AB et O confirmant les fréquences trouvées dans les études marocaines antérieures, et que le Maroc est plus proche de point de vue antigène D de la race africaine que de la race caucasienne [6].

Pour l'âge, on a trouvé que les taux élevés du vWF sont occupés par 83,2% de la population en avançant dans l'âge. Ce qui concorde avec la littérature. Par ailleurs, le taux de vWF varie avec l'âge avec une diminution progressive de la naissance à l'âge de 1 an puis une élévation régulière [1]. Pour un ratio TCA normal (≤ 1,2): On note la présence d'une seule patiente avec un taux bas en vWF. Cependant, 17,9% des taux de vWF sont dans les normes et 81,8% sont supérieurs à la normale. Pour un ratio pathologique (> 1,2): 12,5% avec un taux diminué, 18,8% dans les normes et 68,8% se sont révélés avec un taux élevé à la valeur normale. Donc pour des taux bas en vWF ont des TCA allongé à cause du rôle du vWF qui est la molécule chaperonne du FVIII. Pour un TCA normal une seule patiente avait un taux bas en vWF, mais cela ne pose pas de contradiction puisque le TCA est raccourcis par l'augmentation des facteurs de coagulation en cette période de grossesse concordant avec résultats de la littérature [2]. Le dosage du fibrinogène n'a révélé aucune valeur inférieure à la normale (2-4g/l). Pour des valeurs normales: 1,7% avaient un taux bas en vWF, 30,0% dans les normes, et 68% avec des taux supérieurs aux valeurs usuelles. Et pour les hautes valeurs en Fg (> 4g/l): 0,8% avaient un taux bas en vWF, 17,9% dans les normes et 81,3% avec des valeurs supérieures aux normes. Nos résultats montrent une élévation parallèle des taux de fibrinogène et des taux vWF. Ces résultats ont été illustrés par la corrélation entre les taux de vWF et de fibrinogène par le test de Pearson qui est utilisé pour étudier la corrélation entre les variables quantitatives avec calcul du facteur r de corrélation. Au total, il existe une corrélation positive entre les taux vWF et les taux de fibrinogène avec un r de Pearson à 0,356, cette corrélation est statistiquement significative avec un p < 0,001. Cela concorde avec la littérature en expliquant l'état inflammatoire et la situation physiologique de la femme enceinte [2].

Toutes les patientes qui ont un taux de FVIII diminué (< 50%), ont un taux bas en vWF. Pour les valeurs normales en FVIII (50-145%): une patiente soit 3,4% avait une valeur diminuée en vWF, 86,2% dans les normes, et 10,3% supérieures aux normes. Pour des taux élevés en FVIII (> 145%): 12,5% dans les normes et 87,5% avec des taux élevés. Nos résultats montrent une nette relation entre les taux de FVIII et de vWF du fait que la baisse des taux de ce dernier influence les taux de FVIII. Il est noté que 92,05% de notre population présentaient des taux élevés en FVIII et 87,5% avaient des taux élevés en vWF. Du même, on confirme ces résultats en étudiant la corrélation entre le vWF et FVIII par le test de Pearson avec calcul du facteur r de corrélation. On a trouvé qu'il existe une corrélation positive entre le facteur VIII et le facteur de vWF avec un r de Pearson à 0,597 d'une façon significative p < 0,001. Cela concorde avec la littérature. vWF circule dans le plasma avec le facteur VIII sous forme de complexe non-covalent. Tout changement dans le niveau de vWF plasmatique est couplé à un changementconcordant dans la concentration plasmatique du facteur VIII [7]. Concernant l'influence du groupe sanguin ABO sur les taux de vWF et FVIII, on constate que les taux de vWF selon les groupes du système ABO varient dans le sens suivant: groupe O < A < B < AB (De même pour le FVIII). Ces résultats ont été obtenus en utilisant une analyse de variance ANOVA, on retrouve une différence statistiquement significative concernant le vWF et le FVIII entre les quatre groupes sanguins: p < 0,001. Après correction de Benferoni on retrouve que le taux de vWF du groupe sanguin O est statistiquement différent des autres groupes (plus faible). De même pour le FVIII, le taux chez le groupe O est plus faible que celui du groupe B, AB et A de façon significative. Ce qui concorde à la littérature expliquant l'influence des gènes dits « modulateurs » [8].

## Conclusion

La distribution physiologique des taux plasmatiques du vWF est large, reflétant la sensibilité de cette glycoprotéine à des facteurs génétiques (cas système ABO sanguin) et environnementaux (cas de grossesse), tout en exposant la vie de certains sujets à des risques hémorragiques en cas de déficit quantitatif ou qualitatif du vWF (MW). Comme confirme notre étude la grossesse normale s'accompagne de modifications majeures de l'hémostase, allant dans le sens d'un état d'hypercoagulabilité, suite à l'élévation de certains facteurs de l'hémostase. Au Maroc, il est avantageux d´investir dans ce contexte des études épidémiologiques qui seront nécessaires afin de connaitre les caractéristiques éventuelles de notre population.

### Etat des connaissances actuelles sur le sujet

- Le vWF est un facteur indispensable à l'hémostase, il intervient dans l'adhésion plaquettaire et le transport du FVIII; sa sécrétion endothéliale est augmentée au cours de la grossesse entraînant des taux qui peuvent décapiter certaines formes de la maladie de Willebrand (type 1);
- Le profil des taux du vFW est influencé par le groupe sanguin ABO; ce taux est plus bas en cas de groupe sanguin O;
- La maladie de Willebrand est la pathologie constitutionnelle la plus fréquente de l'hémostase; son diagnostic s'est considérablement amélioré ces dernières années permettant une prise en charge adéquate des patients atteints notamment des patientes enceintes.

### Contribution de notre étude à la connaissance

- La maladie de Willebrand est une maladie méconnue dans notre contexte; notre étude constitue la première enquête permettant de connaitre la prévalence de la maladie de Willebrand chez la femme enceinte;
- Encourager et faciliter la recherche en améliorant la qualité de vie des femmes enceintes afin d'aider les décideurs à opter des stratégies de prise en charge préventives et thérapeutiques des patientes et de leurs familles;
- Connaitre l'influence des groupes sanguins sur les profils des taux de vWF chez la population marocaine permettant d'améliorer la prise en charge des grossesses à risque en réduisant le taux de mortalité fœto-maternelle par syndrome hémorragique lié à la maladie de Willebrand.

## Conflits d'intérêts

Les auteurs ne déclarent aucun conflit d'intérêts.
